# Indocyanine green *versus* technetium-99*m* for sentinel lymph node biopsy in breast cancer: the FLUORO trial

**DOI:** 10.1093/bjsopen/zraf104

**Published:** 2025-10-15

**Authors:** Chu Luan Nguyen, Jianing Kwok, Michael Zhou, Neshanth Easwaralingam, Jue Li Seah, Belinda Chan, Susannah Graham, Farhad Azimi, Cindy Mak, Carlo Pulitano, Sanjay Warrier

**Affiliations:** Department of Breast Surgery, Chris O’Brien Lifehouse, Sydney, New South Wales, Australia; Department of Surgery, Royal Prince Alfred Hospital, Sydney, New South Wales, Australia; Department of Surgery, The University of Sydney, Sydney, New South Wales, Australia; Department of Breast Surgery, Chris O’Brien Lifehouse, Sydney, New South Wales, Australia; Department of Surgery, The University of Sydney, Sydney, New South Wales, Australia; Department of Surgery, The University of Sydney, Sydney, New South Wales, Australia; Department of Breast Surgery, Chris O’Brien Lifehouse, Sydney, New South Wales, Australia; Department of Surgery, Royal Prince Alfred Hospital, Sydney, New South Wales, Australia; Department of Breast Surgery, Chris O’Brien Lifehouse, Sydney, New South Wales, Australia; Department of Surgery, Royal Prince Alfred Hospital, Sydney, New South Wales, Australia; Department of Breast Surgery, Chris O’Brien Lifehouse, Sydney, New South Wales, Australia; Department of Surgery, The University of Sydney, Sydney, New South Wales, Australia; Department of Breast Surgery, Chris O’Brien Lifehouse, Sydney, New South Wales, Australia; Department of Surgery, The University of Sydney, Sydney, New South Wales, Australia; Department of Breast Surgery, Chris O’Brien Lifehouse, Sydney, New South Wales, Australia; Department of Breast Surgery, Chris O’Brien Lifehouse, Sydney, New South Wales, Australia; Department of Surgery, The University of Sydney, Sydney, New South Wales, Australia; Department of Surgery, Royal Prince Alfred Hospital, Sydney, New South Wales, Australia; Department of Surgery, The University of Sydney, Sydney, New South Wales, Australia; Department of Breast Surgery, Chris O’Brien Lifehouse, Sydney, New South Wales, Australia; Department of Surgery, Royal Prince Alfred Hospital, Sydney, New South Wales, Australia; Department of Surgery, The University of Sydney, Sydney, New South Wales, Australia

## Abstract

**Background:**

Standard sentinel lymph node (SLN) mapping for early breast cancer involves technetium-99*m* (^99m^Tc) lymphoscintigraphy. Indocyanine green (ICG) fluorescence allows real-time visualization of lymphatics and nodes while avoiding radiation exposure and the inconvenience of ^99m^Tc, but its inclusion in international guidelines is not widespread. This study compared efficacy and costs between ICG and ^99m^Tc for axillary SLN lymphatic mapping.

**Methods:**

Patients with early breast cancer and clinically negative axilla who underwent lymphatic mapping with ICG and ^99m^Tc were enrolled in a prospective single-institution single-arm non-randomized trial (2021–2024). Data on the number of SLNs, including metastatic nodes, rate of failed mapping, costs, and the surgeon’s reported ease of mapping with ICG compared with ^99m^Tc were collected. Cost analysis used Medicare item numbers and microcosting.

**Results:**

A total of 305 patients were enrolled, with 637 SLNs sampled. The SLN identification rate was 97.8% (95% confidence interval (c.i.) 96.3 to 98.7%) for ICG and 98.3% (95% c.i. 96.9 to 99%) for ^99m^Tc. The mean(standard deviation (s.d.)) number of SLNs identified with ICG and ^99m^Tc was 2.06 (1.99) and 2.07 (2.02), respectively (*P* = 0.871). Metastatic SLNs were identified in 70 of 305 patients (23.0%), with 83 metastatic SLNs in total. ICG identified 79 of 83 metastatic SLNs (95.2%; 95% c.i. 88.3 to 98.1%) and ^99m^Tc identified 82 of 83 metastatic SLNs (98.8%; 95% c.i. 93.5 to 99.8%; *P* = 0.256). Mean(s.d.) surgeon-reported ease for using ICG and ^99m^Tc, rated used a five-point Likert scale, was 1.67 (0.98) (95% c.i. 1.56 to 1.78) and 1.5 (0.59) (95% c.i. 1.43 to 1.57), respectively (*P* = 0.082). ^99m^Tc cost an additional EUR841 (95% c.i. EUR766 to EUR917) per patient but ICG would require > 35 patients before breaking even with initial outlay equipment costs.

**Conclusion:**

ICG fluorescence performed similarly to ^99m^Tc lymphoscintigraphy and may be less costly over the long term.

## Teaser

This prospective trial demonstrated that indocyanine green fluorescence performed similarly to technetium-99*m* lymphoscintigraphy and was potentially less costly in the long term for axillary sentinel lymph node biopsy in breast cancer.

## Introduction

Standard tracers for lymphatic mapping and sentinel lymph node (SLN) biopsy for early breast cancer include blue dye (BD; patent blue, methylene blue, or isosulfan blue) and radioactive colloid labelled with technetium-99*m* (^99m^Tc)^1,2^. The standard dual technique of BD and ^99m^Tc is highly sensitive, with a SLN detection rate of 96.7% and low false-negative rates^3–8^. The use of ^99m^Tc alone has a detection rate of 96.5%, whereas BD alone has a detection rate of 86.8%^8^. Drawbacks of BD tracer include skin tattooing and hypersensitivity reactions^9,10^, whereas drawbacks of ^99m^Tc include inconvenience to patients, cumulative radiation exposure to healthcare workers, waste disposal, and the requirement for access to a nuclear medicine facility^11–14^. These limitations have led to the development of new techniques, including fluorescence-guided SLN biopsy, which involves real-time fluorescence imaging using a near-infrared camera after subdermal administration of the fluorophore indocyanine green (ICG)^8,15,16^.

However, localization techniques using ICG warrant further investigation. This alternative may have potential as a sole tracer for SLN biopsy by combining the advantages of BD and ^99m^Tc without the disadvantages. This may be more relevant once clinical experience, as part of a dual localization technique, has accrued^17^. In smaller studies, ICG has shown equivalent sensitivity to ^99m^Tc, with SLN detection rates ranging from 93.1 to 100%^15,16,18–21^. More data from prospective trials evaluating its effectiveness, as well as costs, are required.

For a new technology to be adopted into clinical practice, it should ideally be as effective as the current standard and at a reasonable cost. Fluorescence imaging requires additional expenditure, including costs of the ICG drug, dedicated camera, and disposable materials. The use of ^99m^Tc has additional costs from hospital infrastructure to accommodate radioactive materials, specialist staff for lymphoscintigraphy, and patient travel^22^. This study aimed to compare ICG to ^99m^Tc for axillary SLN lymphatic mapping in early breast cancer in terms of the number of SLNs identified, including metastatic nodes, rate of failed mapping, ease of use, and associated costs.

## Methods

### Study design and participants

This was a prospective single-institution single-arm non-randomized trial of patients with early breast cancer undergoing SLN biopsy between April 2021 and June 2024 using ICG fluorescence. An earlier study compared ICG fluorescence with BD, whereas the present study compared ICG fluorescence with ^99m^Tc lymphoscintigraphy^17,23^. Trial participation was approved by the Human Research Ethics Committees (HREC) Sydney Local Health District, Australia (Protocol no. X21-0156 & 2021/ETH00747), and the study was registered with the Australian New Zealand Clinical Trials Registry (ACTRN12621001033831). This study followed STARD, the CONSORT Statement (*Table S1*), and the CHEERS 2022 guidelines^24^.

The inclusion criteria were: female sex, age ≥ 18 years, early breast cancer confirmed by core biopsy and a clinically negative axilla, and scheduled for SLN biopsy with breast-conserving surgery (BCS) or mastectomy. Patients with clinically positive lymph nodes, previous axillary or neoadjuvant therapy, or a known contraindication to ICG (that is, a previous reaction to ICG, iodine allergy, chronic kidney disease stage 3, 4, or 5, or pregnancy) were excluded from the study. Patients meeting the inclusion criteria underwent standard-of-care SLN biopsy with the addition of ICG use.

### Surgical technique

The operations were performed by a single surgeon (SW)with over 10 years of experience performing a high volume of SLN biopsies each year. Indocyanine green fluorescence-guided lymphatic mapping was piloted on 40 patients before the start of this study^25^. Patients eligible for SLN biopsy underwent preoperative lymphoscintigraphy with subdermal/peritumour injection of 15–20 MBq ^99m^Tc the day before surgery. At the time of surgery, after anaesthesia and just before the operation, 1 ml Infracyanine^®^ (25 mg/10 ml; SERB, Paris, France) was injected subdermally into the periareolar area of the breast. Movement of ICG in the lymphatic ducts was facilitated by manual massage. ICG fluorescence was elicited and detected using a near-infrared camera (SPY-PHI; Stryker, Sydney, NSW, Australia). Lymphatic drainage was visualized in real time on a monitor. The fluorescence was followed from the site of injection towards the axilla. SLN mapping and biopsy proceeded through either a BCS or mastectomy incision or an axillary incision.

**Fig. 1:**
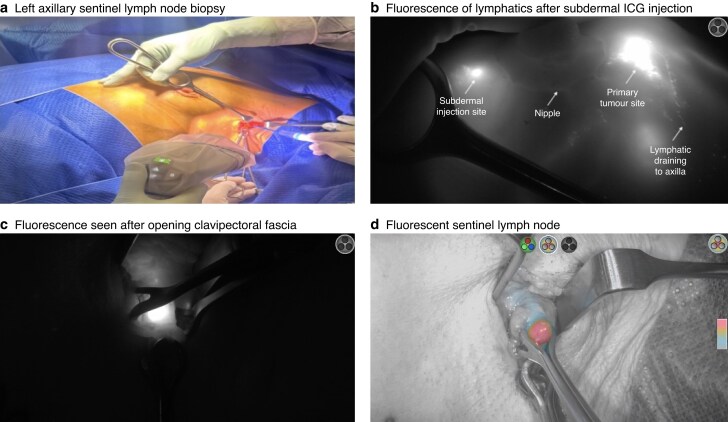
Fluorescence-guided axillary sentinel lymph node biopsy **a** Left axillary sentinel lymph node biopsy with the near-infrared camera set-up, **b** fluorescence of lymphatics observed following subdermal injection of ICG and massage, **c** dissection through the clavipectoral fascia and fluorescence identified within the axilla in fluorescence mode, and **d** a ‘green’ sentinel lymph node retracted out with Babcock forceps demonstrating fluorescence in the colour segmented fluorescence mode. ICG, indocyanine green.

The surgical field was assessed continuously throughout the procedure using the near-infrared camera. The fluorescent lymphatic channels were dissected and followed to the first ICG-avid lymph node. Fluorescent lymph nodes (ICG positive) were then localized and excised (*Fig. 1*), as described previously^26^. These excised ICG-positive nodes were then tested outside the body for radioactivity using a gamma-detecting probe and classified as ^99m^Tc-positive (hot) or ^99m^Tc-negative (Navigator™ 2.0 Wireless; Dilon Technologies, Newport News, VA, USA). SLN removal continued until no residual fluorescence was visible in the axilla. Finally, the axilla was inspected with the gamma-detecting probe to determine whether any radioactivity was left. A SLN was defined as any lymph node exhibiting radioactivity ≥ 10% of the counts measured in the most radioactive (hottest) node, in accordance with established nuclear medicine protocols. This relative threshold allows for the identification of nodes most likely to represent primary lymphatic drainage pathways. All SLNs were sent separately for histopathological analysis. The total number of SLNs identified by ICG, ^99m^Tc, or both was recorded.

### Histopathological assessment

Lymph nodes were serially sectioned at intervals and subsequently stained with haematoxylin and eosin. The pathology report included the number of lymph nodes examined and how many were benign, contained isolated tumour cells (< 0.2 mm in size), micrometastasis (0.2–2 mm in size), or macrometastasis (> 2 mm in size).

### Outcomes

The primary endpoints were the number of SLNs identified, the number of metastatic nodes identified, and the rate of failed mapping with ICG and ^99m^Tc methods separately and using both techniques. Failed mapping was defined as no fluorescent signal or a radioactive signal that was < 10% of the hottest nodal count detected in the lymph node. Secondary endpoints were any complications, costs associated with ICG *versus*  ^99m^Tc methods, and the surgeon's reported ease of identifying SLNs with ICG compared with ^99m^Tc. The surgeon's reported ease of identifying SLNs with ICG *versus*  ^99m^Tc was determined immediately after the procedure using a five-point Likert scale (1 = very easy; 2 = easy; 3 = equivocal; 4 = hard; 5 = very hard). Postoperative information was collected, including complications (such as reactions and anaphylaxis to tracers), as was follow-up visit information up to 90 days after surgery.

### Cost analysis

Cost minimization analysis was used because the outcomes of the alternative technique compared were assumed to be equivalent^27^. The perspective of a third-party payer (Medicare) was adopted. The fixed cost for ICG fluorescence was the purchase price of the SPY-PHI fluorescence imaging system; the variable cost was the cost of each use of the ICG drug. The fixed costs for lymphoscintigraphy were the costs of the gamma-detecting console and probe; the variable cost was the cost of each use of the ^99m^Tc sulfur colloid. Theatre costs were based on microcosting, including costs for personnel and consumables related to the techniques. Additional costs of the lymphoscintigraphy procedure were factored into the calculation. All costs were initially calculated in 2024 Australian dollars, reflecting local context, and subsequently converted to Euros (EUR) to facilitate international comparability.

### Power and sample size calculations

This trial was designed to determine whether ICG fluorescence would be non-inferior to the criterion standard, ^99m^Tc lymphoscintigraphy. The sample size was calculated based on current data in the literature on ICG and ^99m^Tc detection rates^8^. A non-inferiority hypothesis was used, which assumed a SLN identification rate of 96% for each tracer technique and a non-inferiority margin of 6% to obtain an identification rate > 90% (one-sided test significance level α = 0.05). A sample size of 264 patients was required to achieve 80% power to reject the null hypothesis that the SLN identification rate was inferior with ICG relative to ^99m^Tc by a > 6% non-inferiority margin (with a 5% probability of a type I error). At least 290 patients were recruited to account for 10% attrition^17,28,29^.

### Statistical analysis

Continuous variables are presented as the mean with standard deviation (s.d.) or as the median with interquartile ranges (i.q.r.), as appropriate. Dichotomous and categorical data are presented as frequencies with percentages. Concordance rates between the two tracers are presented as frequencies. Fisher's exact test was used for the analysis of categorical variables and *t*-test used for continuous variables. For the primary non-inferiority analysis, a one-sided test with a significance level of α = 0.05 was used. For all other comparisons, two-sided tests were used, and results with *P*≤ 0.05 were considered statistically significant. Multivariate regression was performed if *P* ≤ 0.2 on univariate regression. Statistical analysis was performed with RStudio (version 2024.09).

## Results

### Study population

**Fig. 2:**
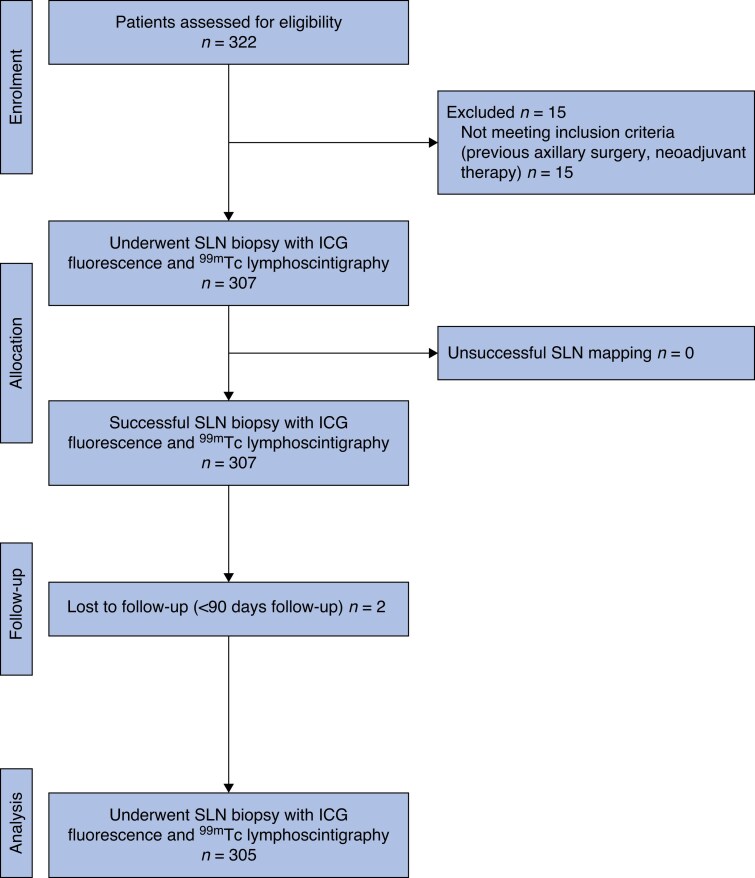
CONSORT diagram and flow chart SLN, sentinel lymph node; ICG, indocyanine green; ^99m^Tc, technetium-99*m*.

A total of 305 consecutive patients who underwent SLN biopsy using both ICG and ^99m^Tc tracers were enrolled in the study (*Fig. 2*). The median age was 62 (i.q.r. 53–70) years and the mean(s.d.) body mass index (BMI) was 26.7 (5.66) kg/m^2^. Most tumours were invasive carcinoma of no special type (77.4%; *Table 1*). ICG fluorescence was well tolerated by all patients, with no ICG-related adverse events identified within the 90-day follow-up period. No allergic reaction, skin necrosis, and long-term skin staining were observed.

**Table 1:** Patient (*n* = 305) and tumour characteristics of the study cohort

**Patient characteristics**	
Age (years), median (i.q.r.)	62 (53–70)
ASA grade, median (i.q.r.)	II (II–II)
BMI (kg/m^2^), mean(s.d.)	26.7(5.66)
**Tumour characteristics**	
Type	
Invasive ductal	236 (77.4%)
Invasive lobular	35 (11.5%)
Mixed/other*	34 (11.1%)
Size (mm), mean(s.d.)	19.31(11.23)
Grade	
1	100 (32.8%)
2	144 (47.2%)
3	61 (20%)
Receptor status	
ER+	253 (83%)
PR+	240 (78.7%)
HER2+	35 (11.5%)
Triple negative	37 (12.1%)
Ki67 index ≥ 14%	132 (43.3%)
Type of operation	
Breast-conserving surgery	240 (78.7%)
Mastectomy	65 (21.3%)

Values are *n* (%) unless otherwise stated. *Includes invasive mucinous carcinoma, micropapillary carcinoma, solid papillary carcinoma, and associated ductal carcinoma *in situ*. i.q.r., interquartile range; ASA, American Society of Anesthesiologists; s.d., standard deviation; BMI, body mass index; ER+, estrogen receptor positive; PR+, progesterone receptor positive; HER2+, human epidermal growth factor receptor 2 positive.

### Lymph node detection rates

A total of 637 SLNs were identified with ICG and/or ^99m^Tc, and confirmed by pathology. There was comparable efficacy between ICG and ^99m^Tc in terms of SLN detection rates. The SLN identification rate for ICG was 97.8% (623 of 637), with a 95% confidence interval (c.i.) of 96.3 to 98.7. For ^99m^Tc, the identification rate was 98.3% (626 of 637), with a 95% c.i. of 96.9 to 99. There were 612 SLNs (96.1%) that were both fluorescent and hot. Eleven nodes (1.7%) were fluorescent only and 14 SLNs (2.2%) only showed ^99m^Tc uptake. The mean(s.d.) number of SLNs identified per patient was 2.06 (1.99) with ICG and 2.07 (2.02) with ^99m^Tc, respectively (*P* = 0.871).

The failed mapping rate was low for both tracers. ICG failed mapping occurred in 14 patients (4.6%), corresponding to a success rate of 95.4% (291 of 305; 95% c.i. 92.4 to 97.2). For ^99m^Tc, failed mapping occurred in 11 patients (3.6%), yielding a success rate of 96.4% (294 of 305; 95% c.i. 93.7 to 98). There was no significant difference in mapping failure rates between the two tracers (*P* = 0.652; *Tables 2*, *3*). The cohort with failed ICG fluorescence mapping had a greater mean BMI than those with successful mapping (30.3 *versus* 26.5 kg/m^2^, respectively; *P* = 0.033). No other variables were found to be significantly associated with failed ICG fluorescence mapping to warrant multivariate analysis (*Table S2*).

**Table 2:** Summary of results of ICG fluorescence *versus*  ^99m^Tc lymphoscintigraphy

	ICG	^99m^Tc	*P*
No. of SLNs*, mean(s.d.)	2.06 (1.99)	2.07 (2.02)	0.871
Failed mapping†	14 (4.6%)	11 (3.6%)	0.652
Metastatic SLNs (83 total)	79 (95.2%)	82 (98.8%)	0.256
Ease of detection‡, mean(s.d.)	1.67 (0.98)	1.5 (0.59)	0.082

Values are *n* (%) unless otherwise stated. *Per patient. †Number of patients. ‡Measured using a five-point Likert scale (1 = very easy; 2 = easy; 3 = equivocal; 4 = hard; 5 = very hard). ICG, indocyanine green; ^99m^Tc, technetium-99*m*; SLNs, sentinel lymph nodes; s.d., standard deviation.

**Table 3:** Number of SLNs identified with ICG fluorescence and ^99m^Tc lymphoscintigraphy

Mapping	^99m^Tc positive	^99m^Tc negative	Total SLNs
ICG positive	612 (96.1%)	11 (1.7%)	623 (97.8%)
ICG negative	14 (2.2%)	0	14 (2.2%)
Total SLNs	626 (98.3%)	11 (1.7%)	637 (100%)

Values are *n* (%). ICG, indocyanine green; ^99m^Tc, technetium-99*m*; SLNs, sentinel lymph nodes.

### Metastatic lymph nodes

Of all 637 nodes biopsied, 83 (13.03%) had metastatic disease. Of the 83 metastatic SLNs, ICG detected 79 (95.2%; 95% c.i. 88.3 to 98.1) and ^99m^Tc detected 82 (98.8%; 95% c.i. 93.5 to 99.8). The concordance between the two tracers was 94% for detection of metastatic lymph nodes. There was no statistically significant difference between the two techniques with respect to the identification of metastatic SLNs (*Tables 2*, *4*).

**Table 4:** Metastatic nodes positive and negative for ICG fluorescence and ^99m^Tc lymphoscintigraphy

Mapping	^99m^Tc positive	^99m^Tc negative	Total SLNs
ICG positive	78 (94%)	1 (1.2%)	79 (95.2%)
ICG negative	4 (4.8%)	0	4 (4.8%)
Total SLNs	82 (98.8%)	1 (1.2%)	83 (100%)

Values are *n* (%). ICG, indocyanine green; ^99m^Tc, technetium-99*m*; SLNs, sentinel lymph nodes.

### Surgeon-reported ease of mapping

There was no statistically significant difference in surgeon-reported ease, using a Likert scale, of mapping SLNs with ICG fluorescence *versus*  ^99m^Tc lymphoscintigraphy, with mean(s.d.) scores of 1.67 (0.98) (95% c.i. 1.56 to 1.78) and 1.5 (0.59) (95% c.i. 1.43 to 1.57), respectively (*P* = 0.082; *Table 2*).

### Cost analysis

The fixed cost for ICG use included the fluorescence imaging system (total, EUR50,006; camera, EUR26,249; console, EUR19,812; software, EUR3946). The fixed cost for lymphoscintigraphy included the gamma-detecting console and probe (total, EUR20,121). Ongoing variable costs were those associated with each vial of ICG drug (EUR71) and ^99m^Tc sulfur colloid (EUR175). Theatre costs were based on microcosting, including costs for personnel and consumables related to both techniques. Ongoing variable costs with lymphoscintigraphy involved the procedure itself, as well as imaging and nuclear medicine physician and technician costs. ^99m^Tc lymphoscintigraphy ended up costing an additional EUR841.35 per patient (95% c.i. EUR766 to EUR917) compared to ICG fluorescence. ICG fluorescence would require > 35 cases before breaking even with initial outlay equipment costs (*Table 5*).

**Table 5:** Costs in Euros of using ICG fluorescence *versus*  ^99m^Tc lymphoscintigraphy for sentinel lymph node biopsy

Item	Cost (EUR)	
	^99m^Tc	ICG
**Initial outlay cost**		
Fluorescence imaging system*		50,005.70
Gamma probe and console†	20,120.53	
**Drugs**		
ICG drug (per vial, 25 mg/10 ml)		70.39
^99m^Tc sulfur colloid (per vial, 10 ml)	175.12	
**Theatre consumables**		
Near-infrared camera disposable sterile cover		3.27
Gamma probe disposable sterile cover	12.06	
**Theatre costs‡**		
Surgeon/anaesthetist fee (per min)	2.00	2.00
Other personnel fee§ (per min)	5.64	5.64
Theatre room fee (per min)	8.91	8.91
**Lymphoscintigraphy (Medicare)**		
Lymphoscintigraphy	196.20	
Ultrasound	57.60	
CT	56.36	
Repeat SPECT/CT	72.71	
**Nuclear medicine costs¶**		
Nuclear medicine physician fee (per min)	1.15	
Other personnel fee# (per min)	1.72	
**Operation (Medicare)**		
Breast-conserving surgery/mastectomy	403.34/456.66	403.34/456.66
Sentinel lymph node biopsy	440.62	440.62
Total cost per patient**	2751.19	1909.84
Total additional cost of ^99m^Tc per patient††	841.35 (766.06, 917.18)	

*SPY-PHI (Stryker, Sydney, NSW, Australia). †Navigator™ 2.0 Wireless (Dilon Technologies, Newport News, VA, USA). ‡Cost per median operation duration of 60 min. §Includes theatre scrub and anaesthetic nursing staff. ¶Cost per median lymphoscintigraphy duration of 2 hours. #Includes nuclear technicians (×3). **Not inclusive of initial outlay cost of imaging system. ††Values in parentheses are 95% confidence intervals. EUR, Euro; ^99m^Tc, technetium-99*m*; ICG, indocyanine green; min, minute; CT, computed tomography; SPECT, single photon emission CT.

## Discussion

In this study, ICG demonstrated non-inferiority to ^99m^Tc with regard to SLN identification. The concordance rate of the two tracers was 96.1%. The detection rate of at least one SLN was 97.8% for ICG fluorescence, compared with 98.3% for ^99m^Tc lymphoscintigraphy. The detection rate of metastatic lymph nodes for ICG and ^99m^Tc was 95.2% and 98.8%, respectively. However, the clinical implications regarding metastatic node detection warrant careful consideration. Confidence intervals around metastatic detection were wider and, although not statistically inferior, the potential for missed metastatic nodes cannot be excluded. Future studies would help to further validate this. Nonetheless, the overall findings support ICG fluorescence as a safe and potentially cost-efficient alternative to 99mTc lymphoscintigraphy for SLN biopsy in breast cancer.

The localization technique using ICG for SLN biopsy in early breast cancer is a relatively novel technology. Dual ^99m^Tc and BD is widely regarded as the standard for SLN mapping. Consensus regarding the role of ICG as a tracer remains unclear. Although US and UK guidelines have yet to include ICG as a recommended option for SLN biopsy, the European Society for Medical Oncology guidelines recognize that, with appropriate training in the dual ^99m^Tc and BD or ICG fluorescence techniques, SLN biopsy can achieve high success rates, with low false-negative rates^30–33^. The most recent guidelines from the Japanese Breast Cancer Society suggest ICG fluorescence as a safe alternative to ^99m^Tc^34^.

In smaller comparative studies, ICG has shown equivalence to ^99m^Tc, with SLN detection rates ranging from 93.1 to 100%^15,16,18–20,35–40^. Equivalent results for ICG fluorescence compared with ^99m^Tc lymphoscintigraphy as a single tracer, and compared with dual-mapping using BD and ^99m^Tc, have been reported in recent meta-analyses^15,29^. However, it can be difficult to draw reliable conclusions from these meta-analyses because the included studies were limited by variable definitions of the SLN and because of the heterogeneity among study protocols, equipment, ICG dosages, and techniques^15,29,41,42^. Most studies were small cohorts or evaluated dual mapping with ^99m^Tc and/or BD, whereas the present study is one of the largest clinical studies available on ICG fluorescence as a single tracer^15,29,41^.

Some institutions support dual mapping over the use of single tracer to increase detection rates and decrease the rate of false negatives^19^. However, the clinical significance of a higher detection rate associated with the addition of another tracer to ICG should be weighed against the potential adverse effects and costs of the additional tracer. Studies have found that the addition of BD as a second tracer may only benefit lower-volume institutions^43^. The present study evaluated the efficacy of ICG fluorescence as a single tracer while using ^99m^Tc as a control in the same patient, potentially reducing bias due to group differences. However, it was not possible to determine false-negative rates^44^. It is very difficult to evaluate ICG fluorescence in the context of axillary dissection given well established evidence that similar oncological outcomes and less morbidity can be obtained from SLN biopsy compared with axillary dissection for clinically node-negative early breast cancer^6,15,44^.

Despite the accuracy of ICG, identifying an excessive number of SLNs has been described in earlier studies as the main concern with ICG fluorescence. This is thought to be due to its low molecular weight and size compared with ^99m^Tc, resulting in rapid migration through lymphatics^29,43,44^. Sampling of excessive nodes during SLN biopsy can increase upper extremity morbidity^45^. In the present study, a mean of two SLNs was sampled, consistent with other studies^44,46,47^. This number was also equivalent to the mean number of lymph nodes excised when using ^99m^Tc alone or in combination with BD^15,29,44^. A relatively smaller dose of ICG (2.5 mg) was used in the present study compared with earlier studies^36^, and improved equipment and protocols over time may have contributed to these findings^29,47^.

Earlier studies reported that another drawback with ICG fluorescence is that the fluorescent signal only penetrates subcutaneous tissue to a depth of up to 2 cm^29,44,47^. SLN detection rates were potentially hampered in patients with a high BMI due to limited emission of the fluorescent signal, with failed mapping associated with BMI > 40 kg/m^248^. Other studies have contradicted this by demonstrating that the SLN detection rate was independent of BMI^16,44^. However, some of these studies included Asian patients, who generally have a lower BMI than other ethnicities, and so the findings may not be generalizable to other populations^16^. In the present study, patients in whom mapping with ICG fluorescence failed had a significantly greater mean BMI than those in whom mapping was successful (30.3 *versus* 26.6 kg/m^2^, respectively). Patient selection based on BMI may play an important role when considering the use of ICG fluorescence.

ICG fluorescence offers convenient administration following general anaesthesia in a similar way to BD. To date, no trials have formally evaluated the learning curve for ICG fluorescence for SLN biopsy in breast cancer^47,49^. The ease of use of ICG fluorescence to identify SLNs in each patient was surveyed in this study and found to be comparable to that of ^99m^Tc. ICG fluorescence combines the advantages of ^99m^Tc and BD by providing the surgeon with dynamic real-time visualization of the lymphatics as the ICG travels from the injection site towards the SLN^18,50^. ^99m^Tc has static feedback because the nodes are detected by audio cues with the gamma-detecting probe, which only provides signal where sufficient tracer has accumulated in SLNs^51^. In the present study, the surgeon found that ICG fluorescence was intuitive to learn and required less navigational skill than ^99m^Tc or BD. The technique may potentially be transferrable to lower-volume institutions.

The cost involved with ICG fluorescence, along with its safety and effectiveness compared with the current standard, is an important consideration in its potential uptake into routine clinical practice. Published cost analyses comparing ICG fluorescence to ^99m^Tc lymphoscintigraphy are lacking^17,22^. The cost-minimization analysis in the present study, although simple, demonstrated that use of ICG fluorescence as a single tracer had an expensive initial outlay cost but would be financially beneficial in the long term due to the significant recurring costs associated with nuclear medicine^29,36^  ^,52^ . ICG is a relatively inexpensive drug compared with ^99m^Tc^15,18,43,53^. The administration of ICG is convenient, because it involves a subdermal injection given during surgery. ^99m^Tc requires lymphoscintigraphy up to 1 day before the operation, resulting in more hospital resources and an increased time burden for the patient^22,51^. The costliest aspect of ICG fluorescence is the initial cost involved with the near-infrared camera, its console, and software^18^. However, the fluorescence imaging system is also used by other specialities at the authors’ institution, such as hepatobiliary, gynaecology and urology, which make the initial investment more justifiable^29,47^. The costs involved with ICG fluorescence may be reduced in the future as near-infrared technology continues to advance and become more widely available^54,55^.

The limitations of this study include that it is a non-randomized study. The results may not be generalizable to other centres because this was a single-centre and single-surgeon study. The cost analysis was limited by the difficulty in accurately estimating travel costs for patients, because ^99m^Tc lymphoscintigraphy requires two travel times, one to reach the nuclear medicine facility and one to reach the theatre for surgery. Such logistical challenges and costs associated with nuclear medicine could be overcome with ICG fluorescence^41,43^. ICG fluorescence offers another tracer option for patients managed in hospitals without access to nuclear medicine, particularly in regional and remote institutions^29,36,41^. The initial outlay costs for ICG fluorescence can include the purchase of a laparoscopic stack, which could further justify its costs because it can be used across other specialties; however, this was not evaluated in the present study. The cost analysis also did not account for a hospital's need to maintain ^99m^Tc for other indications, such as melanoma. Variability in institutional factors, such as staffing, equipment availability, and local practices, may significantly influence the costs associated with ICG, making the results of the present cost analysis less generalizable.

## Supplementary Material

zraf104_Supplementary_Data

## Data Availability

The data that support the findings of this study are available from the corresponding author upon request.
